# Multiscale hierarchical and heterogeneous mechanical response of additively manufactured novel Al alloy investigated by high-resolution nanoindentation mapping

**DOI:** 10.1038/s41598-022-23083-2

**Published:** 2022-10-31

**Authors:** Abhijeet Dhal, Saket Thapliyal, Supreeth Gaddam, Priyanka Agrawal, Rajiv S. Mishra

**Affiliations:** 1 Department of Materials Science and Engineering, University of North Texas, Denton, TX USA; 2 Advanced Materials and Manufacturing Processes Institute, University of North Texas, Denton, TX USA; 3 Present Address: Manufacturing Science Division, Oak Ridge National Laboratory, Oak Ridge, TN USA

**Keywords:** Materials science, Structural materials, Mechanical properties

## Abstract

Smart alloying and microstructural engineering mitigate challenges associated with laser-powder bed fusion additive manufacturing (L-PBFAM). A novel Al–Ni–Ti–Zr alloy utilized grain refinement by heterogeneous nucleation and eutectic solidification to achieve superior performance-printability synergy. Conventional mechanical testing cannot delineate complex micromechanics of such alloys. This study combined multiscale nanomechanical and microstructural mapping to illustrate mechanical signatures associated with hierarchical heat distribution and rapid solidification of L-PBFAM. The disproportionate hardening effect imparted by Al_3_(Ti,Zr) precipitates in the pool boundaries and the semi-solid zone was successfully demonstrated. Nanomechanical response associated with heterogeneity in particle volume fraction and coherency across melt pool was interpreted from nanoindentation force–displacement curves. The hardness map effectively delineated the weakest and strongest sections in the pool with microscopic accuracy. The presented approach serves as a high throughput methodology to establish the chemistry-processing-microstructure-properties correlation of newly designed alloys for L-PBFAM.

## Introduction

The adoption of laser powder bed fusion additive manufacturing (L-PBFAM) is re-scripting the manufacturing paradigm in the aerospace, biomedical, and defense industries. The disruptive ability of this technology stems primarily from the extraordinary design, compositional and microstructural flexibility^1^. However, the L-PBFAM of Al alloys remains challenging due to poor laser absorptivity, high cracking susceptibility, and rapid oxidation tendency of the feedstock^2^. Although Al alloys with eutectic or near-eutectic composition (such as Si-rich Al alloys) have shown appreciable printability, their mechanical properties are not in-line with the industry's expectations^3^. On the other hand, high-strength Al alloys suffer heavily from hot-cracking during the L-PBFAM process^4,5^. One effective mitigation strategy has been carefully selecting alloy composition to improve cracking resistance while achieving the mechanical properties equivalent to or better than that of high-strength Al alloys^6^. Integrated computational materials engineering (ICME)-driven alloy-design approach has recently produced several new printable and high-strength Al alloys^7^. However, to fully harness the commercial potential of these new alloys, minimal use of expensive feedstock material like Sc or inoculated powders (feedstock accounts for roughly 15% of manufacturing cost) and widening the processing window to enhance manufacturing flexibility are necessary^8^.

A novel Al–Ni–Ti–Zr alloy with excellent printability-performance synergy reported by Thapliyal et al*.*^9^ meets these criteria and has potential for widespread industrial adoption. Two important microstructural attributes of the material enable this feat. The first attribute is the delayed solidification of Al-Al_3_Ni eutectic, which minimizes the terminal freezing range and facilitates liquid backfilling at the final stages of solidification (~ 640 °C). This eliminates hot-cracks and enables printing fully dense parts over a wide range of scanning speeds and laser powers. The second factor is a carefully engineered heterogeneous microstructure consisting of multimodal grains, particles, and Al_3_Ni–Al eutectic segregation. This microstructure activates various strengthening mechanisms, enhances work hardening, and provides high strength-ductility synergy in the material. The breakaway from coarse columnar grains typically associated with L-PBFAM and the presence of equiaxed grains is due to the formation of L1_2_ Al_3_(Ti,Zr) particles at an early stage of solidification. These particles provide energetically favorable sites for heterogeneous nucleation (HN) and selective undercooling, engineering ultrafine equiaxed microstructure near the pool boundaries. These ultrafine regions interrupt columnar growth and also help in mitigating hot-cracks. Due to multiple thermal cycles and remelting phenomena during L-PBFAM, a complex level of microstructural heterogeneity and hierarchy is obtained in the final component.

The general practice to evaluate and understand the mechanical properties in such novel Al alloys with complex heterogeneous microstructure has been via traditional macroscale mechanical testing, like tensile and compression testing^10–13^. However, conventional testing methods fail to dissect and delineate mechanisms occurring at various length scales, which are critical to understanding the multifaceted deformation behavior of the L-PBFAM material thoroughly. Chen et al*.*^14^ categorized L-PBFAM induced process stress at three distinctive length scales and concluded that these stresses concomitantly influence mechanical response during tensile loading. Type-I is associated with a long-range stress gradient mainly associated with directional heat flow towards the substrate and multiple heat cycles during L-PBFAM processing. Type-II stresses are in the intergranular or melt pool level length scale. They relate to backstresses arising due to strain incompatibility between the multi-sized grains. Type-III stresses self-equilibrate over a sub-granular length scale and is related to the heterogeneous distribution of dislocation cells, particles, and eutectic segregation.

Furthermore, the macro scale tensile stress–strain curves are significantly influenced by printing defects and fail to delineate the effect of individual entities of the multiscale microstructural hierarchy on the mechanical properties to an appreciable accuracy. The printing defects are also highly stochastic and compromise the repeatability of macro-scale tensile testing^15^. With the rapid evolution of the ICME approach for designing novel alloys for L-PBFAM, a high-throughput testing approach is required to understand the chemistry-processing-microstructure-properties relationships. The present work introduces high-resolution nanoindentation mapping to elucidate complex deformation mechanisms at meso-microscopic length scales within a novel Al alloy specifically designed for L-PBFAM. The results obtained by nanoindentation are impervious to processing defects and evaluate the material's full strengthening potential. So far, the high-resolution nanoindentation mapping has only been limited to distinguishing mechanical responses between phases of varying hardness^16–18^. In this view, the present paper presents a multiscale approach for establishing detailed chemistry-processing-microstructure-properties relationships for additively manufactured novel alloys with complex microstructures and solidification signatures.

## Results and discussion

**Figure 1:**
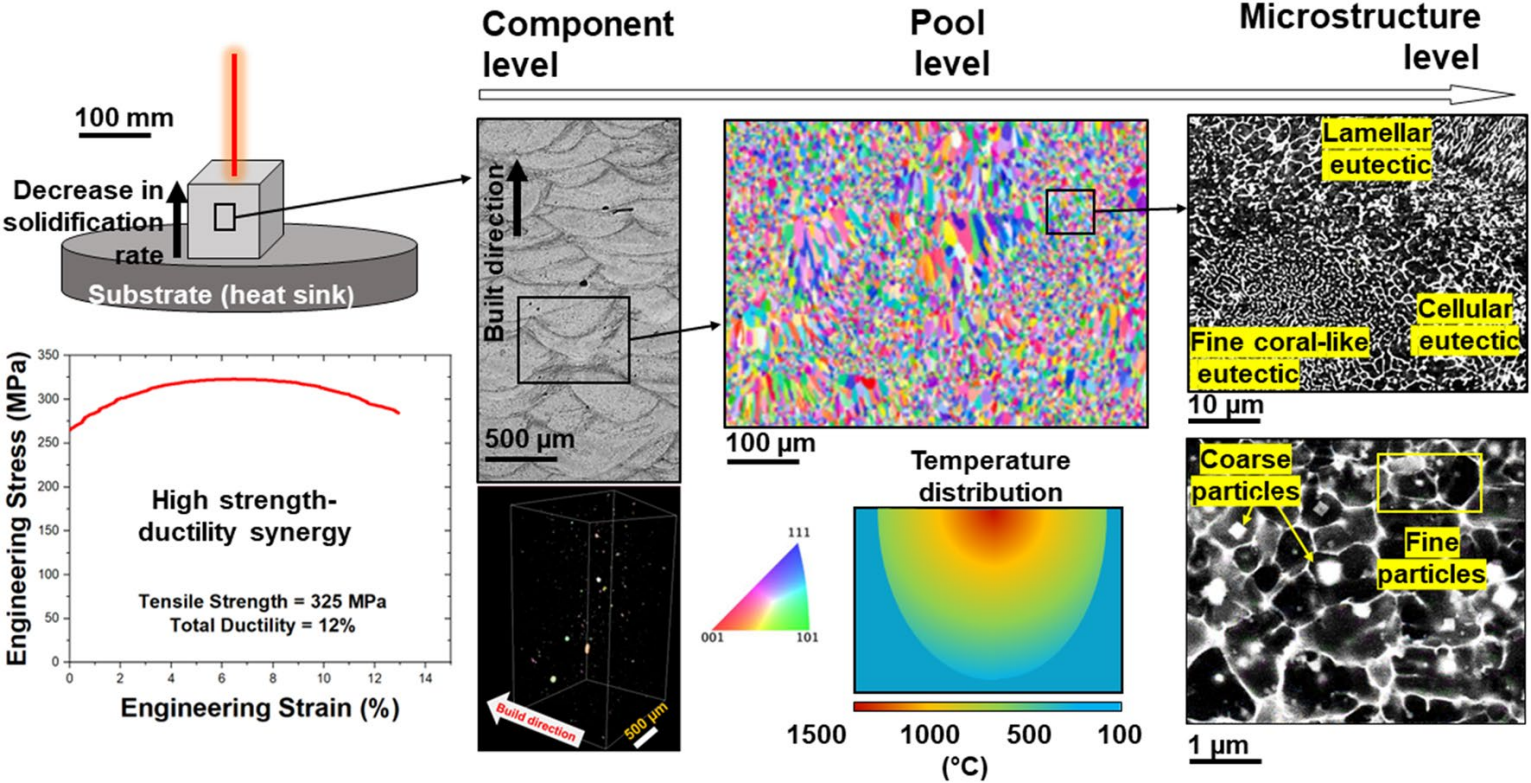
Multiple factors acting at different length scales contribute to the tensile stress–strain response of additively manufactured materials. Long-range thermal stresses and stochasticity of processing defects influence property at the macroscale. Gaussian heat distribution and microstructural heterogeneity impact mesoscopic properties at the pool level. At the microstructural level, heterogeneity in particle and eutectic segregation influences plasticity in micro-nano scales. The presented high-resolution nanoindentation map attempts to investigate properties at the pool and microstructural levels.

Figure 1 provides an overview of the structural heterogeneity present at various length scales in the in the printed Al–Ni–Ti–Zr alloy. Such heterogeneity contributes to the high strength-ductility synergy as observed in the engineering stress–strain curve. At the microstructural length scale, the deformation mechanism is governed by the strain-partitioning between the Al_3_Ni eutectic segregation with the Al matrix and size-dependent coherency or Orowon strengthening effect of Al_3_(Ti,Zr) particles. Backstress strengthening effect^19^ due to multimodal grain size and mesoscale stress-gradient associated with Gaussian temperature profile is expected at the pool level. Large-scale thermal gradients and stochasticity of processing defects significantly influence macroscale mechanical properties. These multiscale mechanisms cooperatively contribute to the high strength-ductility synergy of the L-PBAM Al alloy.

**Figure 2:**
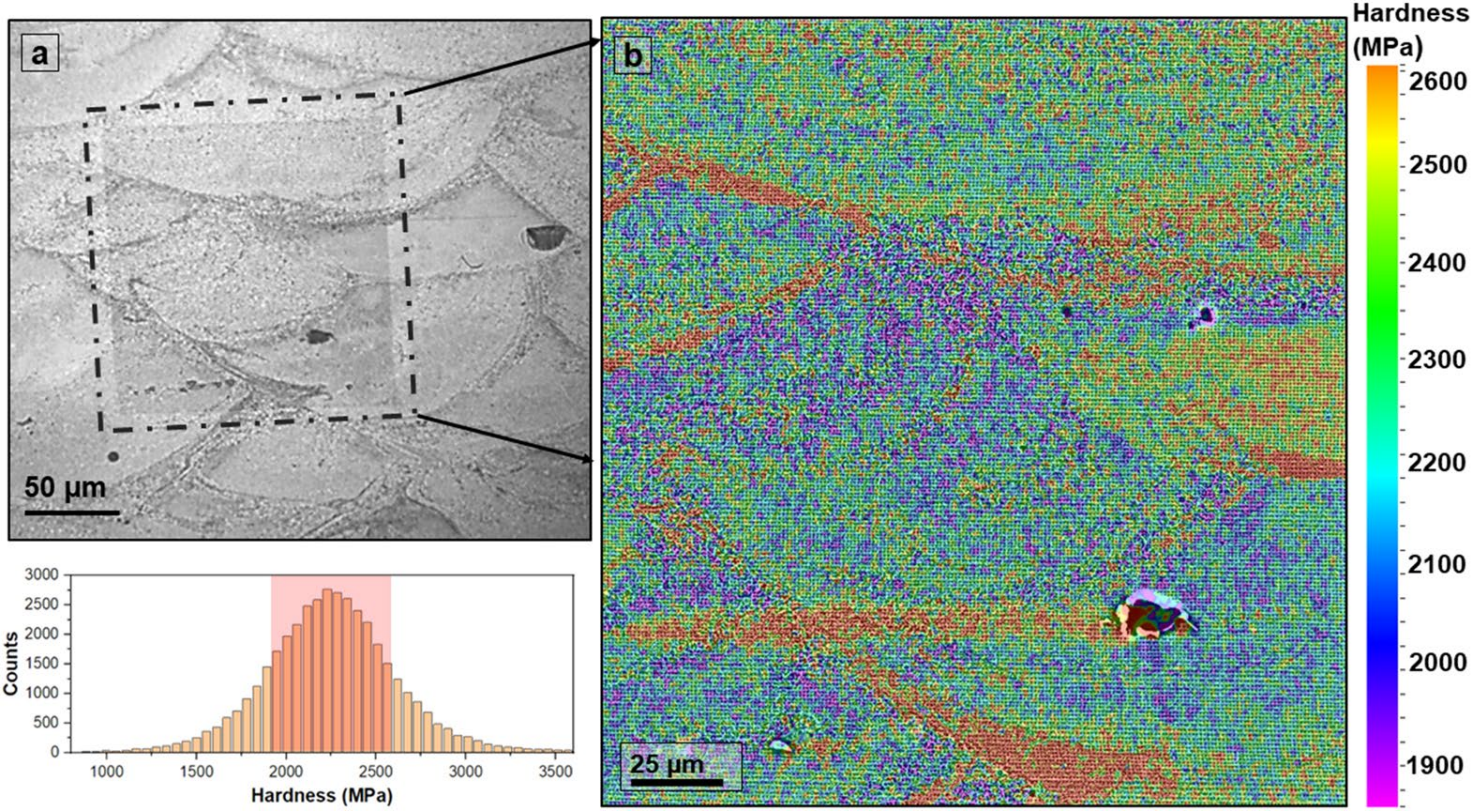
Nano-hardness map covering approximately six melt pools correlates well with the hierarchical and heterogeneous microstructure of the L-PBFAM processed Al–Ni–Ti–Zr alloy. (**a**) The optical micrograph confirms approximately six melt pools in the nanoindented region (dashed box), and (**b**) the hardness map captures the micromechanical variation within multiple melt pools. For statistical accuracy, the data within the one-sigma range was selected for mapping, and the selected region is highlighted in the adjoining histogram.

As evident from the optical micrograph in Fig. 2a, the hardness map covers an area of 150 × 150 µm and contains approximately six melt pools. The micromechanical-microstructural correlation scaling these six melt pools is investigated by analyzing the high-resolution nano-hardness map overlapped with low magnification BSE image (Fig. 2b). To improve the statistical accuracy, data within the one-sigma range were considered for the mapping (highlighted in the corresponding histogram). The hardness map strongly correlates with the hierarchical and heterogenous microstructure observed for this material^9^. Due to the 67° stripe strategy employed in the L-PBFAM process, the captured six melt pools in the hardness map are polar cross-sections of a three-dimensional pool and, therefore, possess unique hardness distribution. However, each melt pool consistently shows a significant hardness variation ranging from 1850 to 2600 MPa. High nano-hardness (> 2500 MPa) is disproportionately observed at the melt pool boundaries (MPB), and these high hardness regions closely follow the distribution pattern of Al_3_(Ti,Zr) particles as observed in the BSE image. The preferential location of particles in the pool boundary is due to the narrow solidification window of Al_3_(Ti,Zr) particles in this alloy. Scheil-Gulliver solidification path^9^ for this material has established that the Al_3_(Ti,Zr) particles (solidification range: 950–650 °C) completely solidify above the melting temperature of pure Al. The Al_3_(Ti,Zr) particles start nucleating at the MPB, and their solidification pattern follows the Gaussian temperature profile. While these particles remain suspended in the liquid Al pool above 650 °C, Marangoni eddies drive a small fraction of particles towards the top end of the melt pool^20,21^. A few particles get trapped in the pool interior during a growth competition event. These trapped particles result in intermittent high hardness responses in the pool interior. Since only a smaller fraction of potent particles are driven to the pool interior, columnar growth is dominant within these regions of the melt pool. Note that the formation of remelting zones also leads to particle dissolution at the pool top and interior^6,9^. A detailed discussion on the effect of remelting zone on microstructure and ensuing mechanical behavior is provided in the subsequent section.

**Figure 3:**
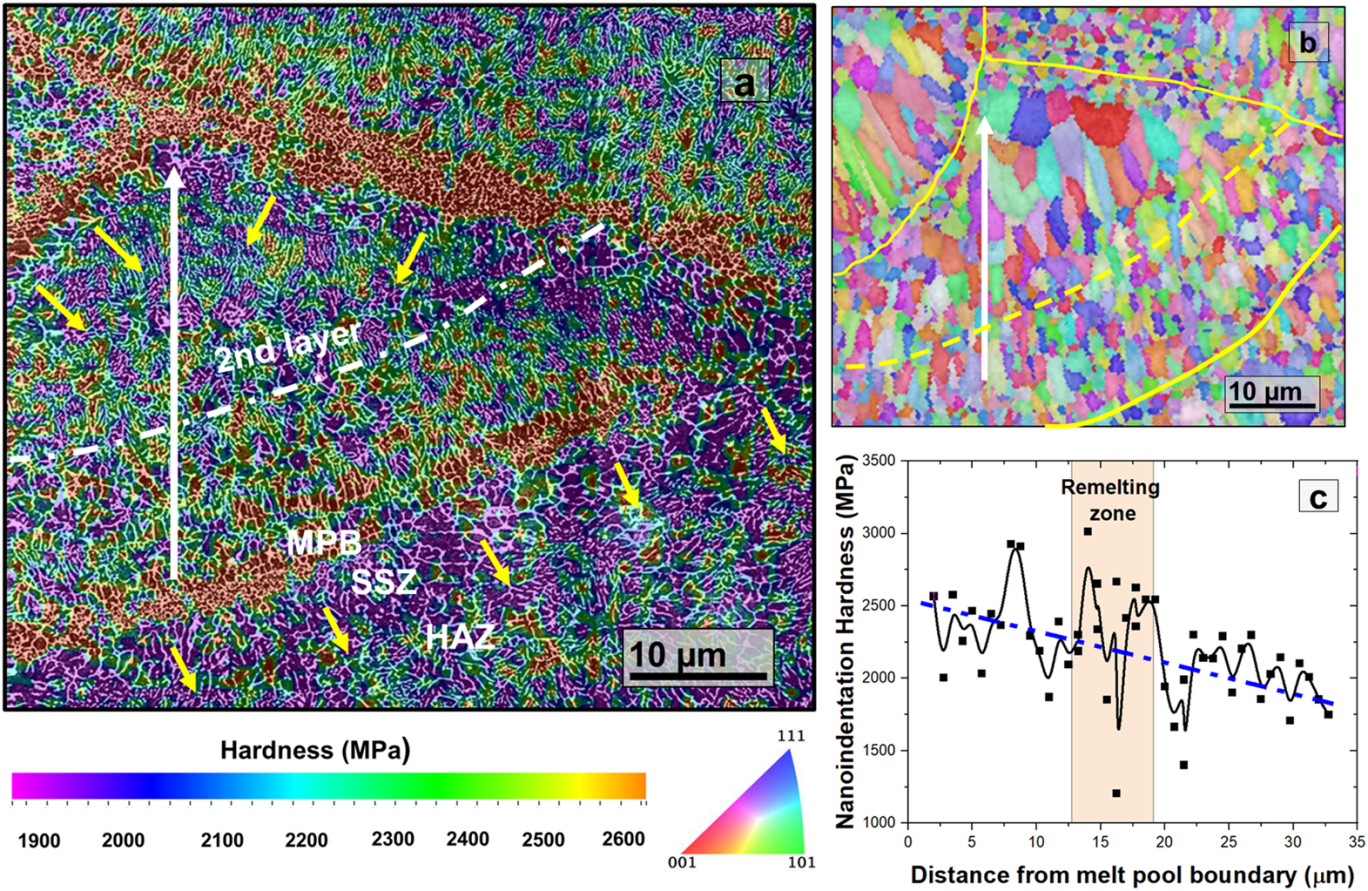
Micromechanical-microstructural correlation within single melt pool (**a**) Hardness map combined with immersion mode BSE image of a single melt pool, (**b**) EBSD map reveals a strong grain size relationship with solidification, and (**c**) nano-hardness trends reveal the influence of complex thermokinetics behavior of L-PBFAM process. The yellow arrows represent the direction of heat extraction from HAZ.

After the primary solidification cycle, the same melt pool undergoes multiple overlapping thermal cycles, which results in hierarchical remelting and solidification sequences during the rest of the L-PBFAM process. The hardness trends inside the pool carry such hierarchical heat distribution signatures. Typically, three distinct thermal zones are formed during these heat cycles: (1) fully remelted zone (FRZ), (2) semi-solid zone (SSZ), and (3) heat-affected zone (HAZ)^22,23^. These heat zones are illustrated in the hardness map of a single melt pool (Fig. 3a). Note that the length scale of SSZ associated with remelting is much smaller than the primary melt pool. In FRZ, the temperature exceeds liquidus temperature (*T*_*L*_), and as the region re-solidifies, the hard Al_3_(Ti,Zr) particles again nucleate and redistribute. They form thinner secondary MPB comprising particle-rich refined grains. These boundaries appear intermittently as hard regions inside the pool. The temperature in SSZ lies between the solidus and liquidus range; thus, complete remelting does not occur in this region. However, the local temperature is high enough to promote significant grain growth (Fig. 3b). The FRZ-SSZ interfaces are susceptible to particle gathering due to Marangoni eddies. Some particles can penetrate through porous mush into the SSZ^24^ or dissolve due to high heat. Depending on the particle fraction and dislocation density, the local hardness in this region is observed to be in the intermediate range (2200–2500 MPa). In HAZ, the temperature remains below the solidus limit. Although not sufficient to melt, the heat in HAZ may over-age, coarsen the strengthening particles, and even promote grain growth, thereby compromising the local hardness. The hardness distribution in HAZ is a thermal trade-off between heat absorbed from the source at the top and the heat rejected to the cold metal at the bottom. Such wide temperature variation results in significant microstructural and mechanical heterogeneity in HAZ. The low hardness regions below the pool boundaries carry strong source-to-sink heat extraction signatures in the hardness map (yellow arrows in Fig. 3a). Median fitting of the nano-hardness data (Fig. 3c) along the direction of the pool reveals a linear decrease in hardness from bottom to top. The primary reason for such a hardness trend is attributed to the variation in cooling rate from top to bottom arising due to an increase in distance from the relatively cooler substrate/layer (heat sink) at the bottom. Intermittent outlying high hardness points are either due to: (1) particle transportation from MPB to pool interior, or (2) retention of few hard Al_3_(Ti,Zr) particles after remelting.

**Figure 4:**
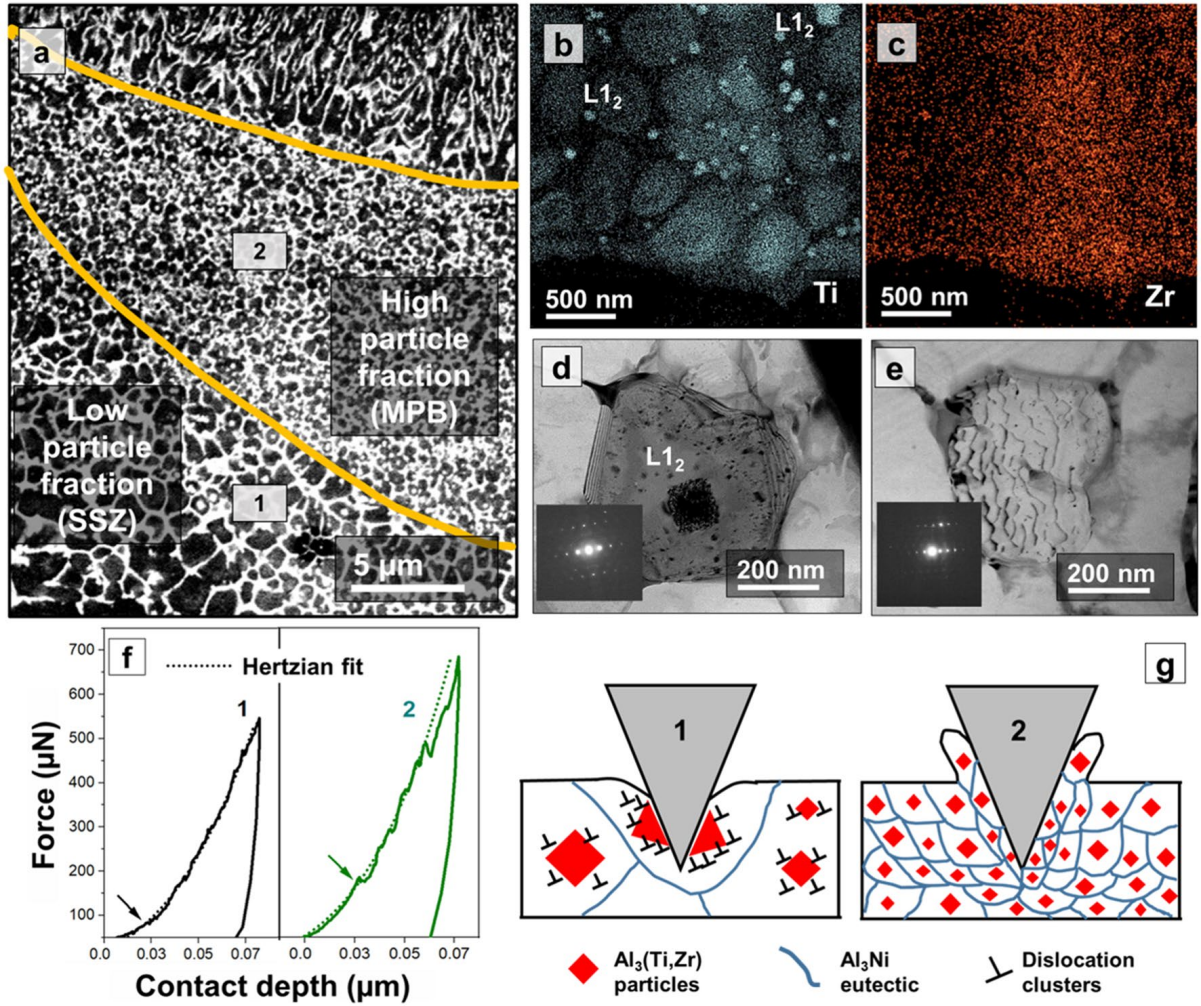
Nanomechanical effect of particle size, coherency, and volume fraction (**a**) The immersion mode high-magnification BSE images captured near melt pool boundary, (**b**, **c**) STEM-EDS maps show variations in particle composition and volume fraction within the pool boundary, (**d**, **e**) high magnification TEM reveals variation in dislocation pattern in particle rich and depleted grains (**f**, **g**) nanoindentation *P–h* curves captures the nanomechanical impact of such heterogeneity in particle distribution, and (**g**) schematics illustrate two possible particle-indenter interactions depending on the local particle size.

The high-magnification BSE micrographs captured around the MPB in Fig. 4a illustrate a wide heterogeneity in particle volume fraction, primarily contributed by convective heat fluxes at MPB. These fluxes redistribute the particles away from the bottom of the MPB and towards the boundary of SSZ^25^. STEM-EDS map indicates a disproportionately higher concentration of coarser Ti-rich precipitates at one end of the MPB (Fig. 4b,c). The compositional variation is due to the hierarchical solidification behavior of the Al_3_(Ti,Zr) precipitates. Depending on their size, coherency, and structure, these particles exhibit either coherency or Orowon particle strengthening. The variation in molecular weight between Al_3_Ti and Al_3_Zr may also influence particle distribution during solidification. The local hardness of the Al_3_Zr and Al_3_Ti phases are 6.32 GPa^26^ and 5 GPa^27^, respectively, significantly higher than pure Al (0.3 GPa^26^). The larger and incoherent L1_2_ particles that facilitate HN also selectively undercool their vicinity^28^, thereby accumulating dense dislocation during solidification (evident in Fig. 4d) and disrupting the solidification-induced dislocation arrangement. This contrasts with the cellular dislocation arrangement in the zone that does not contain these HN-facilitating particles, as shown in Fig. 4e. The dislocation arrangement in this zone corresponds to the traditional solidification-induced dislocation arrangement. However, despite being lean in the fraction of HN-facilitating incoherent L1_2_ particles, a higher concentration of fine coherent L1_2_ particles is observed in this area. Although the fine coherent L1_2_ particles do not facilitate HN, they are very effective strengtheners. These particles are expected to participate actively in dislocation multiplication during the nanoindentation loading stage. On the other hand, pre-existing dislocations around the HN-facilitating L1_2_ particles are expected to reduce the critical stress required for incipient plasticity^29^. Therefore, in both coherent and incoherent forms, the particles in the Al–Ni–Ti–Zr alloy influence dislocation activities during the indentation process. More insights on nanomechanical effect due to heterogeneity in particle distribution are provide in the upcoming section.

Force–displacement (*P–h*) curves (Fig. 4f) capture differences in the nanomechanical response due to heterogeneity in particle distribution. The first pop-in represents a spontaneous dislocation activity under the indenter tip contributed by the incipient plasticity^30^. During plastic deformation, the soft Al matrix cooperatively acts as a shear propagation medium between the hard particles. In SSZ (marked as 1 in the *P–h* curve), the much coarser particles are in a similar length-scale of the indenter tip, resulting in a different nanomechanical response. The first pop-in associated with incipient plasticity occurs at much lower stress than MPB (marked as 2 in the *P–h* curve). This early pop-in is facilitated by the pre-existing dislocation loops concentrated around the incoherent coarse-L1_2_ particles, as described in the previous section. Also, the *P–h* curve does not remarkably deviate from the Hertzian elastic deformation curve, and the non-linear excursions are much smaller than the one observed in the MPB zone. The contributing factor is the larger particle size, which offers high elastic rigidity to the indenter displacement, and the presence of pre-existing dislocation reduces dislocation accumulation capability around the particle. The large rigid particle creates a zone of high stress-localization, eventually leading to particle fracture.

High particle volume fraction, denser grain boundaries, and finer eutectic spacing in MPB intensify the shear localization as they reduce the mean free path of dislocation motion. During the indentation process, the tip frequently interacts with such barriers resulting in frequent pop-in events in the *P–h* curve. A remarkable non-linear excursion follows each pop-in, corresponding to the stress relaxation^31^ after the spontaneous dislocation activity. With the progression of indentation, the intense hydrostatic stress under the indenter tip locally pushes the particle-rich Al matrix. This action continuously reduces the inter-particle distance, i.e., the distance between the hard particles. Eventually, a very dense aggregation of these hard particles and surrounding dislocation clusters leads to a "particle crowding" effect^32^ marked by a very sharp penultimate pop-in event in the *P–h* curve. The non-linear excursion that follows this pop-in corresponds to the intense energy released due to the particle crowding phenomenon. The schematics in Fig. 4g illustrate the two unique indenter-particle interaction mechanisms. Due to particle-indent interaction in the SSZ (predominately elastic deformation), sinking-in of Al surrounding the tip is promoted. Due to fine particle distribution in MPB, significant plastic deformation occurs under the tip. Depending on local work hardening tendency and modulus to strength ratio, either sink-in or pile-up is possible around the tip in MPB. These factors are directly proportional to local volume fraction of the particle. In both scenarios, the particle-rich zone (MPB and SSZ) shows significantly higher hardness than the particle-depleted melt pool interiors, attributed to the described unique particle-indenter interaction mechanisms. Therefore, the particles deployed around the melt pool boundaries are conclusively proven as effective strength/hardness enhancers in the L-PBFAM processed Al–Ni–Ti–Zr alloy.

## Conclusions

The high-throughput nanoindentation investigation successfully delineated various mechanisms occurring at meso and microscopic length scales in the newly designed and laser powder bed fusion additively manufactured Al–3Ni–1Ti–0.8Zr (wt%) alloy. The high-resolution nano-hardness map captures mechanical signatures of hierarchical heat distribution associated with the L-PBFAM process. Microstructural investigation in the intended regions established a strong relationship between the microstructural heterogeneity and the observed mechanical responses. This map is effective in pinpointing the weakest and strongest sections in the pool with microscopic accuracy. The hardness trends across the melt pool are a culmination of varying thermokinetic parameters (due to varying rates of heat transfer towards the substrate) and complex L-PBFAM process dynamics. Al_3_(Ti,Zr) particle formation in pool boundaries provides disproportionately high local hardness. The distribution of these hardening particles is highly heterogeneous across the melt pool due to complex L-PBFAM thermokinetics. Cooperative shear propagation between hard particle and soft Al matrix is possible micromechanism inside melt pool boundary due to the high density of the fine coherent particles. A sharp pop-in event at the penultimate stage of indentation in this zone indicates the massive dislocation activities imparted by the crowding of hard particles within the soft Al matrix. The particle-crowding effect also amplifies the dislocation multiplication phenomenon. Due to a low particle fraction, the deformation behavior is based on site-specific particle-indenter interactions in the semi-solid region. A predominantly rigid body interaction is observed in this zone due to similar length scales of indenter tip and coarse particle. Therefore, this study has led to an understanding of the multiscale deformation mechanisms at the melt pool length scale (meso scale) as well as at the microstructural length scale (nano-micro scale). At the meso scale, such understanding has been enabled by the synergistic use of hardness map spanning multiple pools and BSE and EBSD maps, whereas at the microstructural scale, the synergistic use of site-specific *P–h* curves and high-resolution BSE and TEM micrographs elucidate the chemistry-processing-microstructure-properties relationships for the novel Al–Ni–Ti–Zr alloy.

## Materials and methods

### Additive manufacturing

The methods involved in L-PBFAM processing of the Al–3Ni–1Ti–0.8Zr (wt%) alloy have been described in detail in our previous work^9^. The specimen processed at a laser power of 350 W, scanning speed of 1400 mm/s, hatching distance of 0.13 mm, and layer thickness of 0.03 mm were used for nanomechanical and microstructural characterization throughout this study.

### Thermal modeling

A standard temperature spatial map considering a pseudo-stationary Gaussian heat source was determined using Rosenthal solution within a single melt pool^33^. The following material properties^34^ were used for the model: density = 2.7 g/cm^3^, specific heat = 0.963 J/g °C, thermal conductivity = 117 W/mK, absorptivity = 0.65. MATLAB was used for numerical modeling.

### Nanoindentation

FemtoTools FT-NMT04 nanomechanical testing unit equipped with Berkovich tip and operating in continuous stiffness measurement mode was employed. A fused Si standard was used for tip area calibration, and a nominal tip diameter of 15 nm was calculated. Indentation size effect was abated, and a steady hardness reading was obtained after a depth of 20 nm for the fused Si silica standard. Nanoindentation mapping was done in displacement-controlled mode with a maximum depth of 70 nm. An ultra-fine spatial resolution of 750 nm was adopted to cover an area of 150 µm × 150 µm, giving 40,000 indents. The loading and unloading times of 2 and 1 s were selected, and the sampling rate was fixed at 100 Hz to get significant data points for the loading and unloading curves. Oliver-Pharr's method was adopted for hardness measurement^35^. A small coupon measuring 5 mm × 5 mm × 3 mm (length × width × height), was cut out from the printed block by using Buehler IsoMet high precision cutting saw. Before carrying out the nanoindentation, the sample was meticulously polished using a series of ultrafine emery papers followed by cloth-polishing using a 0.02 µm colloidal silica suspension in Allied Multiprep semi-automatic precision polisher.

### Post-indent microscopy

Low magnification images of the indented region were captured using a tabletop optical microscope. Then the same area was rigorously investigated under an FEI Nova Nano scanning electron microscope (SEM). Backscattered electron (BSE) images were captured in immersion mode to visualize segregation and precipitation distribution in the indented region. Electron backscattered diffraction (EBSD) mapping using Hikari Super EBSD detector was performed to visualize other aspects of the microstructure, such as grain shape, size, and orientation. Site-specific transmission electron microscopy (TEM) and scanning TEM electron dispersive spectroscopy (STEM-EDS) was performed using the FEI Tecnai G2 TF20 microscope operating at 200 kV. Foil for the TEM study was prepared using FEI Nova 200 dual-beam focused ion beam microscope.

## Data Availability

All data generated or analyzed during this study are included in this published article.
